# Metabarcoding dietary analysis in the insectivorous bat *Nyctalusleisleri* and implications for conservation

**DOI:** 10.3897/BDJ.11.e111146

**Published:** 2023-11-14

**Authors:** Sarah J. Bourlat, Martin Koch, Ameli Kirse, Kathrin Langen, Marianne Espeland, Hendrik Giebner, Jan Decher, Axel Ssymank, Vera G. Fonseca

**Affiliations:** 1 Leibniz Institute for the Analysis of Biodiversity Change - Museum Koenig, Bonn, Germany Leibniz Institute for the Analysis of Biodiversity Change - Museum Koenig Bonn Germany; 2 Department of Biogeography, University of Trier, Germany Department of Biogeography University of Trier Germany; 3 Bundesamt für Naturschutz, Bonn, Germany Bundesamt für Naturschutz Bonn Germany; 4 CEFAS, Weymouth, United Kingdom CEFAS Weymouth United Kingdom

**Keywords:** bat conservation, Chiroptera, diet analysis, metabarcoding, prey source habitats, Vespertilionidae

## Abstract

In this study, we aim to uncover diet preferences for the insectivorous bat *Nyctalusleisleri* (Leisler's bat, the lesser noctule) and to provide recommendations for conservation of the species, based on the analysis of prey source habitats. Using a novel guano trap, we sampled bat faeces at selected roosts in a forest in Germany and tested two mitochondrial markers (COI and 16S) and three primer pairs for the metabarcoding of bat faecal pellets.

We found a total of 17 arthropod prey orders comprising 358 species in *N.leisleri* guano. The most diverse orders were Lepidoptera (126 species), Diptera (86 species) and Coleoptera (48 species), followed by Hemiptera (28 species), Trichoptera (16 species), Neuroptera (15 species) and Ephemeroptera (10 species), with Lepidoptera species dominating in spring and Diptera in summer. Based on the ecological requirements of the most abundant arthropod species found in the bat guano, we propose some recommendations for the conservation of *N.leisleri* that are relevant for other insectivorous bat species.

## Introduction

Bats play an important role in pest control, seed dispersal and pollination (Kunz et al. 2011, Baroja et al. 2019), but are threatened by the loss of foraging habitat and insect declines (Tiede et al. 2020). Determining the prey spectrum and identifying suitable foraging areas is thus key to making conservation decisions in habitats occupied by insectivorous bat species. In addition, dietary variation in bats has been shown to be dependent on landscape and agricultural practices (Aizpurua et al. 2018); therefore, changes in land use can lead to the loss of foraging habitat, as well as source habitats suitable for the whole life cycle of prey species. The importance of extending bat conservation areas beyond directly-used hunting grounds to include prey source habitats has been highlighted (Arrizabalaga-Escudero et al. 2015) and particular attention should be paid to cover all habitats for arthropod prey species that have several life stages, as many arthropods are known to show ontogenetic habitat shifts (Carr et al. 2020, [Bibr B10260170][Bibr B10260170], Carr et al. 2020This is also highly relevant for bat protection under the EU Habitats Directive (92/43/EEC), where protected sites should include not only roosts, but habitats necessary for the entire life cycle of bats.

*N.leisleri* is a bat of 13-18 g and a wingspan of 26-32 cm (Dietz et al. 2009). This bat is a small member of the genus *Nyctalus* with a western Palaearctic range (Europe and north-west Africa) with scattered records in the eastern Palaearctic (Pakistan, Afghanistan, the Himalayas) (Juste and Paunović 2016). In late summer and autumn, large parts of the European population migrate south to spend the winter under milder conditions in southern France, Spain and Italy (Boston et al. 2021). Mating happens during late summer on or prior to the beginning of migration. After hibernation and spring-migration, the bats return to their summer habitats and the females form nursery colonies, mainly in tree cavities (Boston et al. 2021), but also in buildings behind wall sidings. They have long, narrow wings adapted for fast flying speeds and for catching insects during flight in the open airspace above the canopy and water bodies, as well as near street-lights and forest edges (Boston et al. 2021). The Conservation status on the IUCN Red List, as well as the European Red List is LC: Least Concern (European Commission et al. 2007, Juste and Paunović 2016).

Current threats to *N.leisleri* include: 1) the reduction in insect abundance due to increased pesticide use; 2) changes in land use leading to the disappearance of fallow land, permanent grassland, hedges and margins, causing the loss of insect-rich habitats; 3) habitat loss due to the draining of wetlands and water bodies in forests and open countryside; 4) habitat degradation through reduction of natural or semi-natural forests; 5) the loss of old trees with high roost potential; 6) renovation work on buildings leading to loss of roosts and roosting opportunities and 7) wind-energy development due to direct collision with rotor blades especially during migration (Boston et al. 2021).

Bats prey upon a wide variety of arthropod species of various sizes, diurnal and nocturnal and flying or non-flying, but many studies find that Lepidoptera, Diptera and Coleoptera represent the dominant prey orders (Alberdi et al. 2012, Arrizabalaga-Escudero et al. 2015, Baroja et al. 2019, Tiede et al. 2020, Alberdi et al. 2020. Leisler's bat is an insectivorous aerial hawker known to catch insects in flight in the open air space above the forest canopy and close to forest edges, some of which are caught in swarms (Waters et al. 1995, Kaňuch et al. 2005). Radio tracking shows that *N.leisleri* commutes to foraging sites up to 13.4 km away from the roost (Shiel et al. 2006a). Based on visual analysis of taxonomically-informative remains found in faecal pellets of *N.leisleri*, the most frequently encountered prey were from the insect orders Lepidoptera, Diptera, Coleoptera and Trichoptera (Beck 1995, Kaňuch et al. 2005, Waters et al. 2006). The presence of Trichoptera in the diet indicates that *N.leisleri* hunts over water bodies (Beck 1995, Vaughan 1997, Shiel et al. 2006b). However, soft-bodied prey species may be underestimated using this method and taxonomically-important parts of prey species may be missing (Beck 1995, Alberdi et al. 2012). While most dietary studies focus on the analysis of faecal pellets, no high-resolution molecular studies of *N.leisleri* diet exist to date. High resolution dietary analyses in bats can help answer a wide variety of ecological questions, such as the relationship between dietary niche breadth and spatial distribution (Alberdi et al. 2020) or help shed light on bat foraging ecology (Alberdi et al. 2012).

Direct observation of feeding is generally very difficult in nocturnal bats and visual identification of prey arthropod remains in bat faeces does not generally result in taxonomic identification of the prey below order or family level (Alberdi et al. 2012). DNA metabarcoding has revolutionised the field of dietary analysis, revealing much higher prey diversity than previously recorded through morphological analysis in many taxa, ranging from bats (Clare et al. 2009, Zeale et al. 2010, Bohmann et al. 2011, Tiede et al. 2020), to fish (Leray et al. 2013, Jakubavičiūtė et al. 2017, Bourlat et al. 2021) and even invertebrates (Waldner and Traugott 2012, Sint et al. 2015). In general, primers covering a wide range of taxa are used for gut content analysis, where shorter fragments of 100 - 250 bp of the mitochondrial cytochrome c oxidase I (COI) gene are generally sufficient to provide taxonomic resolution at the species level (Meusnier et al. 2008, Zeale et al. 2010). This is advantageous for amplification from dietary remains, where DNA is expected to be highly degraded. Molecular methods enable prey identification up to species level, which can be very useful for addressing questions relating to specific features in the prey species, such as wingspan, whether they are tympanate species or whether they are nocturnal or diurnal species. In addition, foraging habitats can be inferred from the consumed prey species using finer scale taxonomic resolution of prey allowing the broadening of the scope of ecological studies on bats (Alberdi et al. 2012), such as their role in providing important ecosystem services (e.g. pest control) in agricultural landscapes (Baroja et al. 2019).

In this study, we sampled bat droppings (guano) at the roost of *N.leisleri* in a natural forest reserve in North Rhine-Westphalia in Germany during March to September 2017. There were three defined objectives to our study. First, to provide a high-resolution analysis of arthropod prey species and seasonal trends in the insectivorous bat species *N.leisleri*. Second, to compare the performance of two fragments of varying lengths for COI, the 313 bp ‘mini barcode’ (mlCOIintF combined with dgHCO2198, hereafter COImldg) (Meyer 2003, Leray et al. 2013) and the 157 bp fragment (ZBJ-ArtF1c combined with ZBJ-ArtR2c, herafter COIArt) (Zeale et al. 2010) and a 110 bp region of the mitochondrial 16S gene (IN16STK-1FW combined with IN16STK-1Rv, herafter 16S) (Kartzinel and Pringle 2015) in the identification of arthropod species from bat faeces. Third, to identify the ecological requirements for some of the prey species identified and, based on this, make recommendations for the conservation of *N.leisleri* prey habitats.

## Data resources

The data underlying this study have been submitted to the NCBI SRA archive under accession number PRJNA752700.

## Material and methods

**Research site**

Sampling was carried out in the EU Natura 2000 site 'Waldreservat Kottenforst' (DE5308303), located near Bonn, Germany between 180 and 200 m above sea level. The forest has an area of 2450 ha and is dominated by sub-atlantic and medio-atlantic oak (*Quercusrobur*, *Quercuspetraea*) and oak-hornbeam (*Quercus* sp., *Carpinusbetulus*) forest, partially with varying admixture of beech (*Fagussylvatica*). Hydromorphic soils with high water tables provide numerous small water bodies. The forest is managed for wood production and includes "wilderness areas", corresponding to unmanaged stands. To the north and east, the forest borders urban areas of the city of Bonn and the highly urbanised and industrialised Rhine valley. To the north-west, the Kottenforst is connected to the Waldville forested area and west to southeast, the forest borders agricultural areas.

**Capture and radio-tracking of bats**

Bats were radio-tagged between 2014 and 2016 to find roost trees within the Natura 2000 site, in order to align management decisions with nature conservation goals. Bats were caught with mist nets at small water bodies and at potential foraging sites. Suitable animals (female, not pregnant, no injuries, minimum average weight) were equipped with transmitters (Telemetrie-Service Dessau) weighing between 0.3-0.5 g (Permission number: RSK 67.1-1.03.20-18/14-M). Tags were attached between the scapulae using surgical glue. Roost trees were tracked down the day after tagging and checked for the presence of bats for the next 10 - 14 days until transmitters fell off or the transmitter battery was consumed. For this study, bat guano was sampled at the main roost tree of *Nyctalusleisleri*, which was identified in 2014 in a woodpecker cavity at a height of 9 m. This roost tree was used constantly during the summer months for several consecutive years.

**Bat guano sampling**

**Figure 1.**
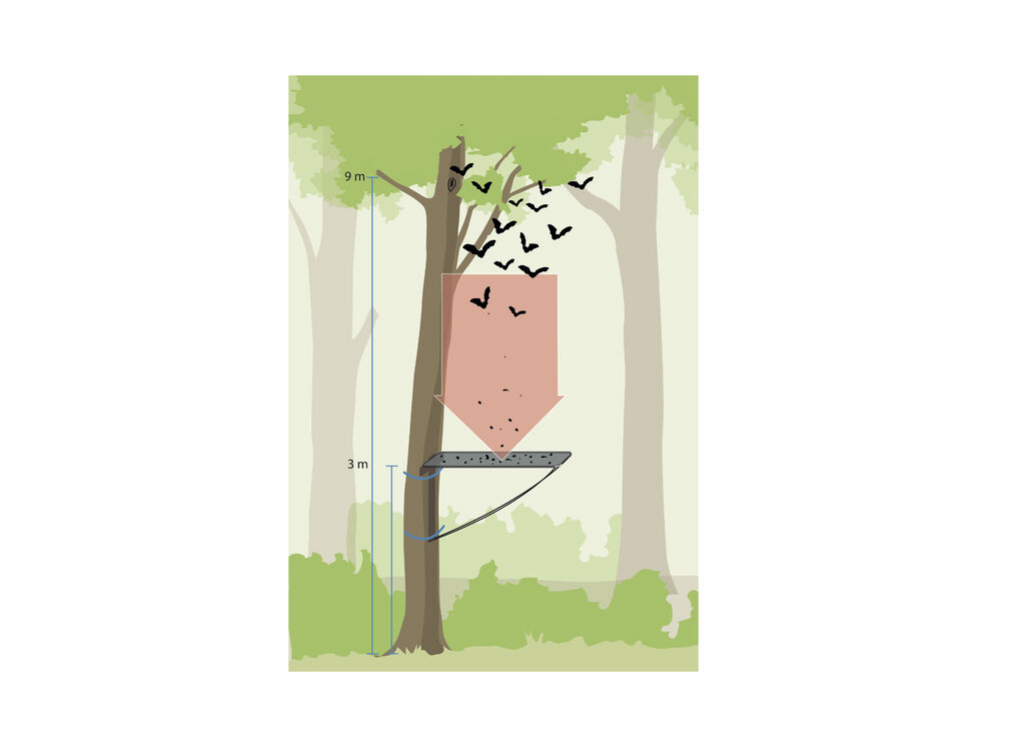
Design and installation of the guano trap (3 m) and roost entrance (9 m).

A novel type of guano trap was installed beneath the roost entrance (Fig. 1). The guano trap is a lightweight rectangular frame of PVC-pipes (25 mm in diameter) with a mosquito-net (mesh width 1.4 mm), attached to the trunk of the roost tree at 3.5 - 4 m height, approximately 3 to 6 m below the roost entrance. The catchment-area of the trap is 2.2 m^2^.

Returning bats show pre-dawn swarming behaviour at occupied roost-trees (Naďo and Kaňuch 2013, Stanton 2016). The bats fly in close circles around the roost tree, land and leave in close proximity to the roost entrance (‘touch and go’) (Kaňuch 2007, Naďo and Kaňuch 2013) and stick guano pellets on the trunk close to the roost entrance. It is assumed that dawn swarming is part of group decision-making and day roost selection of the colony (Schöner et al. 2010, Chaverri et al. 2018). Since bats have a rather low digestive efficiency (Barclay et al. 1991) and a short retention time of prey remains in the digestive system (Roswag et al. 2012), they frequently drop faeces pellets which can be caught by the guano trap.

The trap was checked after nights when swarming was likely to happen. It was assumed that good conditions for swarming were warm nights, with no wind and no rain in the second half of the night. During unfavourable weather conditions, the trap was checked on a regular basis every 2-3 days to remove leaves, small twigs and other debris. Pellets were collected from the net, stored in 15 ml sterile sampling tubes and dried with silica gel and/or stored in 2-propanol. This sampling method is non-invasive and bats do not have to be caught or disturbed to collect faeces for dietary analyses. However, pellets collected can originate from different individuals and possibly even different bat species, due to interspecific swarming behaviour at the roost. Therefore, species identity of the bats was checked using both COI and 16S primers upon library sequencing and data analysis. After species identity check, nine samples confirmed to be from *N.leisleri* were included for further analyses. All samples of bat faeces collected and analysed in this study are detailed in Suppl. material 1.

**DNA extraction and amplicon library preparation from bat faeces**

DNA was extracted from bat guano pellets using the Zymo Quick-DNA™ Fecal/Soil Microbe Midiprep kit, following the manufacturer’s instructions. Guano pellets stored in ethanol were first dried and approximately 40 mg of guano were subsampled from the pellet pool for DNA extraction. All samples (stored in ethanol and silica) were extracted in three replicates including a negative control consisting of sterile water. DNA concentration was measured using the Quantus™ Fluorometer with the QuantiFluor® dsDNA System (Promega). All samples were diluted to 2 ng/µl.

PCR amplification was performed using a 2-step PCR approach. The first PCR was carried out in a total volume of 15 µl per replicate, using 7.5 µl of Q5 Hot Start High ‐ Fidelity 2X Master Mix (NEB), 0.5 µl of each primer (10 µM), 0.5 µl of Bovine Serum Albumin (Thermo Fisher Scientific), 5 µl Sigma H_2_O and 1 µl of DNA. PCR1 conditions involved denaturation at 98°C for 2 min, followed by 20 cycles at 98°C for 40 sec, 50°C for 40 sec and 72°C for 30 sec and a final extension step at 72°C for 3 min. DNA extraction negative controls and PCR negative controls (water) were included for every PCR reaction.

PCR1 products were purified using the HT ExoSAP-IT^TM^ (Thermo Fisher Scientific), with 4 µl ExoSAP for 15 µl PCR 1 product, following the manufacturer’s protocol.

In a second PCR step, the Illumina index adaptors were attached to the purified PCR1 product, which was split into two tubes, each with 7 µl of PCR1 product. Amplifications were carried out in a total volume of 25 µl with 12.5 µl of Q5 Hot Start High ‐ Fidelity 2X Master Mix (NEB), 1.2 µl of each primer (10 µM), 1 µl of Bovine Serum Albumin (Thermo Fisher Scientific), 2 µl Sigma H_2_O and 7 µl PCR 1 product. PCR2 conditions involved a denaturation at 98°C for 2 min, followed by 20 cycles at 98°C for 40 sec, 55°C for 30 sec and 72°C for 30 sec and a final extension step at 72°C for 3 min.

All replicate PCR2 products were pooled, visualised by electrophoresis on a 2% agarose gel (120 V 20 min, 150 V 40 min, 450 mA, 150 W) and purified with the QIAquick gel extraction kit (Qiagen). All purified PCR products were then diluted to the same concentration (3 ng/µl) and pooled into two amplicon libraries. One library comprised the 313 bp COI fragment and the second library the 157 bp COI and 110 bp 16S fragments.

**Sequencing**

The purified amplicon library pools were sequenced on four runs on the Illumina MiSeq platform (2 x 300 bp) using the v.2 Chemistry at the Centre for Genomic Research (CGR, Liverpool University).

**Bioinformatic methods**

Data sequenced at the Centre for Genomic Research (Liverpool, UK) had already undergone a first quality check. The raw fastq files were trimmed for the presence of Illumina adapter sequences using Cutadapt version 1.2.1 (Martin 2011). Sequences were further trimmed using Sickle version 1.200 (Joshi and Fass 2011) with a minimum window quality score of 20. Reads shorter than 20 bp after trimming were removed. Only sequences passing this first quality check were available for download from the CGR server. The downloaded sequences were checked for the presence of the three primer pairs using Cutadapt version 2.10 with Python 3.6.10 (Martin 2011) with the following settings: maximum error rate (-e): 0.1, minimum overlap (-O): 20, minimum sequence length (-m): 150. Each primer-pair dataset was analysed separately. Only sequences with both forward and reverse primers were retained for further analysis. The primers were removed from the sequences before being uploaded to the QIIME2 pipeline (Bolyen et al. 2019). For denoising using Dada2 (Callahan et al. 2016), sequences were truncated to the following lengths: forward and reverse reads of COI mIdg to 175 bp and 170 bp, respectively; forward and reverse reads of COIArt to 216 bp and 169 bp, respectively; forward and reverse reads of 16S to 126 bp and 126 bp, respectively.

Depending on marker, two different reference databases were used. COI sequences were blasted against the German Barcode Of Life (GBOL) database, downloaded from (https://doi.org/10.20363/gbol-20210128) on 29 January 2020 using the following settings: (a) 'query coverage high-scoring sequence pair percent' (-qcov_hsp_perc) was set to 90, meaning that a sequence was reported as match when 90% of the query formed an alignment with an entry of the reference file; (b) minimum percent identity (-perc_identity) was set to 97, requiring the reference and query sequence to match by at least 97% to be reported as a match. The format of the output file was customised using the –outfmt settings ‘6 qseqid sseqid pident’. Taxonomic assignment with the GBOL database yielded 36 arthropod species for mldg and 241 arthropod species for COIArt in the nine guano samples from *N.leisleri* (Suppl. material 2).

The mitochondrial 16S sequences were blasted against a customised 16S reference database downloaded from NCBI GenBank on (29 December 2020). The following search parameters were applied:16S[All Fields] AND (animals[filter] AND is_nuccore[filter] AND mitochondrion[filter] AND ("100"[SLEN] : "1000"[SLEN])). Taxonomic assignment with the GenBank database using a 97% blastID yielded 119 arthropod species for the nine guano samples from *N.leisleri* (Suppl. material 2).

**Figure 2.**
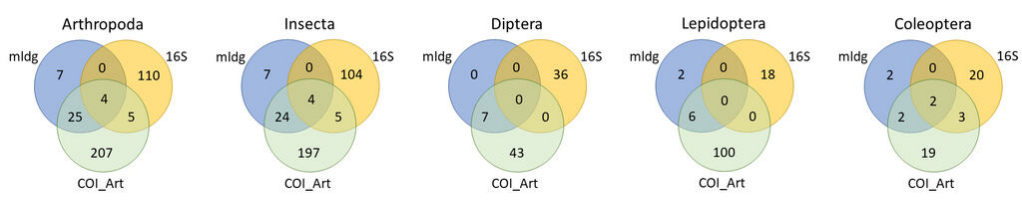
Venn diagrams showing species overlap of different markers (COImldg, COIArt, 16S) for guano samples of *N.leisleri* for Arthropoda, Insecta, Coleoptera, Diptera and Lepidoptera.

**Figure 3.**
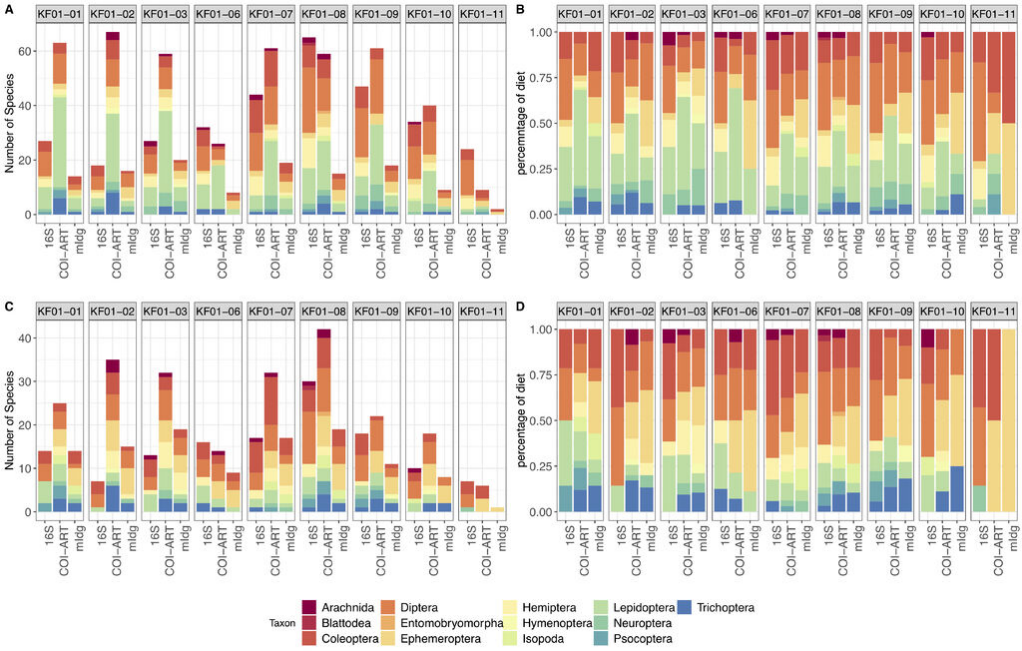
**A** Number of species detected per arthropod order in the guano samples at roost KF01 depending on marker and primer pair. **B** Relative number of species per arthropod order in the guano samples at roost KF01 depending on marker and primer pair. **C** Number of species detected per arthropod order in the guano samples at roost KF01 depending on marker and primer pair (rarefied dataset). **D** Relative number of species per arthropod order in the guano samples at roost KF01 depending on marker and primer pair (rarefied dataset). For this analysis, the ASV table was converted to a presence/absence matrix and read counts were not taken into account.

**Figure 4.**
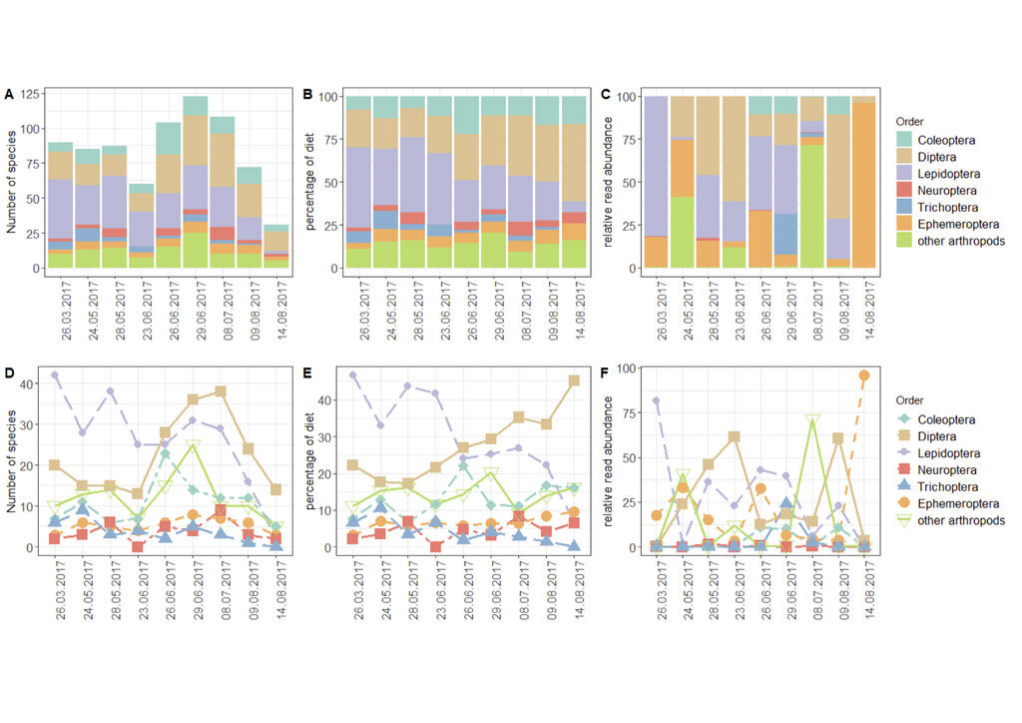
Timeline showing arthropod community composition at order level in the guano of *N.leisleri*, all three markers combined (COImldg, COIArt, 16S). With the exception of plots showing RRA assigned to major groups depending on sampling date (4C and 4F), read counts were not taken into account. **A, D** Number of species of each arthropod order detected at each time point; **B, E** Relative number of species per arthropod order as a percentage of the diet; **C, F** Species detected in each arthropod order, based on relative read abundances.

For the ecological analyses, ASV tables converted to a presence/absence matrix were uploaded into R studio (version 1.4.1106; R version 4.0.4.). For statistical analysis, nine guano samples, assigned uniquely to *N.leisleri* with a 100% Blast match, were analysed (KF01-01, KF01-02, KF01-03, KF01-06, KF01-07, KF01-08, KF01-09, KF01-10, KF01-11). Venn Diagrams were prepared using the package VennDiagram (version 1.6.20) (Chen and Boutros 2011). Assessed community composition depending on markers was visualised using the R packages ggplot2 (version 3.3.3., Wickham (2016)) and RColorBrewer (version 1.1-2, Neuwirth (2011)). For visualisation of *N.leisleri* diet over time, the R packages ggplot2 (version 3.3.3.) and ggpubr (version 0.4.0.Kassambara (2018)) were used. As it has previously been shown that diet analyses based on presence/absence data are very conservative and sometimes overestimate the food consumed in small quantities, we show in parallel analyses based on relative read abundance (RRA) calculated using the formula of Deagle et al. (2018). RRA was calculated for all arthropod and lepidopteran taxa (Suppl. material 3, Suppl. material 4). For calculation of the RRA per sample, the number of reads assigned to each arthropod taxon within a sample was divided by the sum of the of reads for all arthropod taxa in that sample and multiplied by 100 ((number_of_reads_per_taxon_and_sample / total_number_of_reads_per_sample)*100). For calculation of the total RRA in all samples combined, the sum of the reads assigned to a taxon across samples was divided by the sum of the reads for all arthropod taxa in the dataset (all samples combined) and multiplied by 100 ((Number_of_reads_per_taxon / total_number_of_reads)*100). Corresponding R code for figures 2, 3 and 4 can be found in the supplementary materials (Suppl. material 5).

Taxonomy assignment for the 16S mitochondrial marker was carried out against the NCBI database. This database is more incomplete than the GBOL database for the arthropods, especially arthropod species from Germany. These assignments are more likely to represent the best available match and are, therefore, likely biased at the species level. Based on this, we excluded taxonomic assignments with 16S from the analysis based on RRA.

**Molecular identification of species occupying the roosts**

All bat species identification was confirmed using both COI and 16S primers upon library sequencing and data analysis, since guano provides a non-invasive source of DNA that includes information from the bat as well as dietary items, parasites and pathogens (Swift et al. 2018). When taxonomy assignment for each guano sample retrieved exclusively *N.leisleri* with 100% BLAST match, we could confirm that the pellet originates from *N.leisleri* and the sample was included for further analysis (Table 1, samples marked in bold).

## Results

Denoising with Dada2 yielded 1519 ASVs (amplicon sequence variants) for COI mIdg, 1107 ASVs for COIArt and 565 ASVs for 16S for the samples included in our analysis (KF01-01, KF01-02, KF01-03, KF01-06, KF01-07, KF01-08, KF01-09, KF01-10, KF01-11). Taxonomic assignment with the GBOL database yielded 36 arthropod species for mIdg and 241 species for COIArt for the nine guano samples (Suppl. material 2). Taxonomic assignment with the GenBank database using a 97% blastID yielded 119 arthropod species for 16S (Suppl. material 2).

### Molecular identification of species occupying the roosts

The bat species occupying each roost were checked by molecular identification of the bat droppings upon library sequencing and data analysis (see Methods section), confirming the presence of *N.leisleri* exclusively with 100% BLAST match in nine of our samples (KF01-01, KF01-02, KF01-03, KF01-06, KF01-07, KF01-08, KF01-09, KF01-10, KF01-11). Presumed bat species occupying the roosts, based on radio tracking, were mostly, but not always identified accurately, with additional bat species sometimes detected (e.g. in samples KF01-05 and KF03-01 where *Myotisbechsteinii*, *Myotisnattereri*, *Plecotusauritus* and *Myotismystacinus* were detected in addition to *Nyctalusleisleri*) (Table 1). Samples KF01-04, KF01-12 and KF02-02 were removed from subsequent dietary analyses due to contamination from the yellow-necked mouse *Apodemusflavicollis* (presumably due to its ability to climb trees and access the guano traps). All samples from KF03 and KF02 were removed because the presumed bat species were not found in the samples and KF01-05 and KF03-01 were removed because additional bat species were found in addition to *N.leisleri*.

### High-resolution analysis of arthropod prey species in N.leisleri guano and comparison of different markers

The most species-rich arthropod orders found in the nine samples of *N.leisleri* guano for all markers combined (COImldg, COIArt and 16S) were Lepidoptera (126 species), Diptera (86 species) and Coleoptera (48 species), followed by Hemiptera (28 species), Trichoptera (16 species), Neuroptera (15 species) and Ephemeroptera (10 species). Other less species-rich orders (with less than 10 species) were the Araneae, Psocoptera, Hymenoptera, Opiliones, Entomobryomorpha, Ixodida, Isopoda, Blattodea, Lithobiomorpha and Siphonaptera (Table 2).

The most efficient marker in terms of arthropod species detection from bat guano for all samples combined was the COIArt marker with 241 arthropod species overall in contrast to the mldg marker (36 species) or the 16S marker (119 species). The same pattern was observed for the class Insecta and the arthropod orders Lepidoptera and Diptera (with 230, 106 and 50 species detected, respectively with COIArt). For the Coleopterans, similar numbers of species were detected with the COIArt and the 16S marker (26 and 25 species, respectively) (Fig. 2).

The number of arthropod species recovered per sample also varied depending on the primer pair used, but overall, the COIArt primer pair proved to be most effective (Fig. 3A). The relatively small size of the fragment (157 bp) means this primer works particularly well for highly-digested gut contents (Zeale et al. 2010). The taxonomic composition of the arthropod prey found in *N.leisleri* faeces was similar for all samples at roost KF-01. Prey composition showed a majority of Lepidoptera species, followed by Diptera, Coleoptera and Hemiptera in most of the samples, as well as Ephemeroptera dominating in some of the samples (KF01-011) and Trichoptera, Neuroptera, Psocoptera, Entomobryomorpha and Arachnida making up a minority of species in most of the samples (Fig. 3B). The observed taxonomic composition pattern was similar without rarefaction (Fig. 3A and B) or with rarefaction of the ASV tables (Fig. 3C and D).

### Seasonal trends in prey consumed

The overall number of species found in the bat guano was between 60-100 species from the end of March to the end of June, reaching a peak at the end of June and beginning of July and declining rapidly at the beginning of August (< 75 species) to the middle of August (< 40 species) (Fig. 4A). Arthropod community composition found in the guano at order level varied according to time of sampling and season, with more species of Lepidoptera consumed through March to June and declining at the beginning of July (Fig. 4D), when Diptera replace Lepidoptera in their relative contributions as percent of species in the diet (Fig. 4B and E). However, declines in species numbers are observed for all insect orders from the beginning of July, indicating that this is a general trend (Fig. 4D). Other insect groups consumed throughout March to August include the Coleoptera, Ephemeroptera and, to a lesser extent, Neuroptera and Trichoptera (Fig. 4A and B). Analysis of the species detected in the guano, based on relative read abundances, showed a completely different pattern with no clear trend, with Lepidoptera dominating in the March sample and Ephemeroptera dominating in the mid-August sample (Fig. 4C and F).

### Most abundant species in the bat guano

The 25 most abundant Lepidoptera species found in the bat guano, based on RRA for the COI marker, ranged from 27.6% (*Cydiafagiglandana*) to 0.05% (*Agriopisleucophaearia*) of total lepidopteran reads across all analysed samples. Other species included: *Apameaunanimis* (21.7%), *Xanthorhoeferrugata* (15.6%), *Hypenaproboscidalis* (8.6%), *Axyliaputris* (6.4%), *Cnephasiaasseclana* (6.1%), *Dioryctriaabietella* (2.9%), *Eupsiliatransversa* (2.7%), *Oligiaversicolor* (2.6%), *Peridromasaucia* (1.8%), *Mimastiliae* (0.9%), *Ochropleuraplecta* (0.7%), *Polypogontentacularia* (0.5%), *Xestiac-nigrum* (0.2%), *Lomaspilismarginata* (0.2%), *Apameamonoglypha* (0.2%), *Mythimnaalbipuncta* (0.1%), *Sideridisreticulata* (0.1%), *Phlogophorameticulosa* (0.07%), *Peribatodesrhomboidaria* (0.07%), *Callitearapudibunda* (0.06%), *Oligiafasciuncula* (0.06%), *Mamestrabrassicae* (0.06%), *Subacronictamegacephala* (0.06%) and *Agriopisleucophaearia* (0.05%) (Suppl. material 3).

Based on abundance information (average RRA across all samples), the 20 most abundant arthropod species found in the bat guano, based on the COI marker, were four species of Ephemeroptera: *Ephoronvirgo* (13.2%), *Ephemeradanica* (11.6%), *Caenishoraria (2.8%)*, *Baetisfuscatus* (2.3%), nine species of Lepidoptera: *Cydiafagiglandana* (12.9%) , *Apameaunanimis* (10.2%), *Xanthorhoeferrugata* (7.3%), *Hypenaproboscidalis* (4.0%), *Axyliaputris* (3.0%), *Cnephasiaasseclana* (2.8%), *Dioryctriaabietella* (1.4%), *Eupsiliatransversa* (1.3%) and *Oligiaversicolor* (1.2%), one species of Trichoptera: *Lepidostomahirtum* (9.0%), three species of Diptera: *Fannialeucosticta* (2.3%), *Tipulalunata* (1.8%) and *Cheilotrichiacinerascens* (1.0%), two species of Coleoptera: *Lagriahirta* (3.4%) and *Haploglossamarginalis* (1.1%) and one species of Heteroptera: *Troilusluridus* (0.9%) (Suppl. material 4). For most of these species, we summarised wingspan, larval food, flying time, number of generations and habitat, in order to derive a set of recommendations for their conservation (Table 3).

## Discussion

Guano samples provide a non-invasive source of DNA that includes information from the bat, but also dietary items, parasites, and pathogens (Swift et al. 2018). In this study, our analyses confirmed the presence of five different bat species (*Myotisnattereri*, *Nyctalusleisleri*, *Plecotusauritus*, *Myotismystacinus* and *Myotisbechsteinii*), with multiple species sometimes found at the same roost. The latter can occur since bats eavesdrop on other bat species to find roosts in forests (Jones 2008, Ruczyński et al. 2009). In particular, Schöner et al. 2010 showed that *M.bechsteinii*, *M.nattereri* and *P.auritus* can approach bat boxes with played-back bat calls. We assume that bat calls, even from other species, were a cue for *N.leisleri* to approach and find possible roosts within our study area.

### Dietary analyses and seasonal trends

Our study reveals that *Nyctalusleisleri* feeds on a wide range of arthropods comprising 358 species, with the most diverse orders being the Lepidoptera (126 species), Diptera (86 species) and Coleoptera (48 species), followed by Hemiptera (28 species), Trichoptera (16 species), Neuroptera (15 species) and Ephemeroptera (10 species). Based on read abundance data, our study shows that *Nyctalusleisleri* feeds primarily on Lepidopteran and Ephemeropteran taxa, mainly nocturnal insects including pest arthropods that infest forest trees. *N.leisleri* showed the behaviour of a generalist forager, switching between prey according to seasonal availability; however, our results should be interpreted with caution due to the small sample size analysed here and possible inter-individual variability.

The most speciose arthropod prey group detected was the Lepidoptera with 125 species detected. Most of these belonged to the night active families Noctuidae, Tortricidae and Geometridae (Suppl. material 3), which was expected given most insectivorous bat species feed on night‐flying insects (Kolkert et al. 2019), but might also be explained by the relatively large size of moths from these families (Table 3), which makes them particularly rewarding for bats. The second most species-rich arthropod order in the bat guano was the order Diptera, with 86 species detected, followed by the Coleoptera with 48 species detected. The dominant prey orders Lepidoptera, Diptera and Coleoptera found in this study have also been observed in previous insectivorous bat dietary studies (Alberdi et al. 2012, Arrizabalaga-Escudero et al. 2015, Baroja et al. 2019, Tiede et al. 2020, Alberdi et al. 2020). One should note here that the Lepidoptera, Diptera and Coleoptera are amongst the most speciose insect orders, which might also explain some of the patterns observed here.

Patterns of prey switching have been observed in bat populations (Waters et al. 1995, Kaňuch et al. 2005) and this study was no exception. In fact, the timeline of prey detected in the bat guano revealed a switch in the dominating prey species from Lepidoptera to Diptera, which coincided with the presumed birth of the young bats at the end of June or the beginning of July. A similar pattern showing a peak of dipterans during the month of July has also been observed in a recent metabarcoding dietary study of the greater horseshoe bat, but this varied amongst colonies observed and according to their surrounding landscapes (Tournayre et al. 2020). It is known that parturition induces a switch in foraging behaviour of *N.leisleri* (Shiel et al. 2006a). Foraging flights are shortened and distances flown from the roost are reduced due to regular nursing constraints. The opportunistic *N.leisleri* then preys on insects found in close proximity to the roost tree and is most active during this period. The reduction in Lepidoptera species found in the bat guano from mid- to end of June might reflect late flying Lepidoptera species emerging later in the year, mirroring a phenology shift away from midsummer (Fox et al. 2021). This may result in an abundance gap of Lepidoptera in June/July since the early moths do not fly anymore and the late moths have not yet emerged. An opportunistic bat species, such as *N.leisleri* can switch prey and make use of the next abundant insect species. The timing of predator demand and prey availability is crucial (Thackeray et al. 2016, Bell et al. 2019) and the effects of changes in phenology across trophic levels of insects and bats are not well understood and may have an impact on non-opportunistic bat species.

According to Deagle et al. (2018), converting sequence read counts to occurrence information can introduce strong biases and is not always the most conservative approach, so in addition, we decided to analyse relative read abundances (RRA) to obtain a semi-quantitative estimate of the prey species found. Interestingly, while the most species-rich orders found in the bat guano, based on presence/absence information, were the Lepidoptera, Diptera and Coleoptera, our analysis based on RRA showed that the Ephemeroptera were also amongst the dominating insect orders. Whilst we are aware that read counts can be unreliable, RRA summaries can be a valuable addition and can, in some cases, provide a more accurate view than diet summaries based on occurrence information only, as long as they are interpreted carefully (Deagle et al. 2018). Our analysis of RRA across samples, based on the COI marker, showed that two species of ephemeropterans (*Ephoronvirgo*, *Ephemeradanica*) dominated in some of the samples, indicating that *N.leisleri* is able to take advantage of large ephemeropteran swarms. Various orders including Chironomidae, Trichoptera and Ephemeroptera feature mainly aquatic larvae and their imagines do not go far away from their place of origin next to waters. These groups also form large swarms during the adult phase, allowing bats to catch them easily (Beck 1995), which highlights the importance of preserving water bodies as prey source habitats for *N.leisleri*.

### Ecological requirements of the prey species

Many of the insects identified in the guano as bat prey are known to display unique ecological characteristics (see Table 3 for larval food, flying time, number of generations and habitat). The most abundant species found, the ephemeropteran *E.virgo*, forms large swarms over the lower stretches of rivers and the nymphs burrow in the sediments. It was extirpated from the Rhine and its tributaries in the early 20th century due to deteriorating water quality and only reappeared in the late 1980s once the water quality improved (Kureck and Fontes 1996). Thus, for several decades, one of the currently most abundant food sources was entirely absent from the study area, suggesting an opportunistic shift back to this food source once it became available again. The other aquatic insects found in the guano have similar life histories and also swarm, but are mostly associated with smaller rivers and streams or standing water.

Most of the Lepidoptera and Diptera species are common species found in a wide variety of habitats (Table 3) and some lepidopterans are considered pests, such as *Cydiafagiglandia* (especially on chestnut, for example, Pedrazzoli et al. 2012, and *Dioryctriaabietella* on conifers (Svensson et al. 2017). Since these pest species were found in the guano, *N.leisleri* could be potentially important in controlling the populations of these species, as has been shown before in other bat species with agricultural pests (Aizpurua et al. 2018).

### Recommendations for the management of N.leisleri

As apex-predators for the insect fauna in European landscapes, bats provide crucial ecosystem services, such as pest control (Fenton 1997, Altringham 2011, Aizpurua et al. 2018) and valuable information as indicator species on the ecological quality of the landscape (Jones et al. 2009). Several key objectives identified to protect bat species include roost protection in forests (e.g. Müller and Bütler (2010)), roost protection in caves and buildings (Voigt et al. 2015, Medellin et al. 2017), landscape connectivity (Threlfall et al. 2012, Frey-Ehrenbold et al. 2013, Kalda et al. 2015, Carlier et al. 2019), light pollution (Stone et al. 2015, Laforge et al. 2019) and, more recently, insect population decline (Arrizabalaga-Escudero et al. 2015, Carr et al. 2020). The less specific the ecology of a species is, the more difficult it is to formulate conservation measures. For *N.leisleri*, many of the generalist approaches in bat conservation may work. Colonies will benefit from an increase in roosting opportunities in forests due to more variety in roosts with microclimatic fingerprints and less competition from other tree cavity dwelling animal species. High flying bats, such as *N.leisleri*, may bridge disrupted landscape connectivity, are not displaced by artificial light and are even able to exploit insect assemblages at street lights (Mathews et al. 2015, Russo et al. 2018).

However, the loss of insect biomass in open landscapes and forests (Hallmann et al. 2017, Seibold et al. 2019) will affect an opportunistic insect predator like *N.leisleri*. Many of the target prey species (Lepidoptera, Ephemeroptera) are attracted by artificial light sources, which can cause a population to decline (van Grunsven et al. 2020). To reduce negative impacts on insects and bats, light use should be re-assessed especially close to forest edges (Rowse et al. 2015). It is well established that insect diversity is closely related to management intensity (Leidinger et al. 2019). Coniferous, plantation-like stands with a low age gradient in trees support less insect species than a deciduous forest with a diverse age spectrum. Older forest stands with higher biomass also support a higher biomass of herbivorous insects (Leidinger et al. 2019). This information should be considered, especially when reforesting drought-related clear cuts in our research area, the Kottenforst. Planting a high diversity of deciduous trees will support insect diversity in the future and, keeping the old forest stands with high biomass will support insect biomass today. Additionally, the maintenance and management of meadows and forest road margins have a great impact on the insect fauna (Arrizabalaga-Escudero et al. 2015). Extensive, simultaneous mowing will demolish the food source vegetation for oviposition of insects. Rotational systems which leave part of the grassland or forest borders unmown every year would support overwintering and survival of above-ground immature insect stages of many Lepidoptera and Diptera. Similarly, aquatic insects are also an important food source not only for *N.leisleri*, but support many other bat species (Heim et al. 2018, Ancillotto et al. 2019). Our results show that the ephemeropteran *E.virgo* is one of the main food sources for the aerial hawker *N.leisleri*. This species, as well as other aquatic species, rely on clean, chemically unstressed water bodies and streams. The preservation of rivers, streams and ponds benefits the ecological productivity of a landscape and supports an opportunistic species such as *N.leisleri*.

## Conclusions

In this study, we show that metabarcoding has the capacity to improve the quality and resolution of ecological data, such as diet and prey data, which can be a turning point for the success of habitat and conservation management measures. From the *N.leisleri* prey data obtained, we derive a set of key recommendations for *N.leisleri* habitat and conservation management:

- Preserve rivers, streams and ponds (Ephemeroptera, Trichoptera, Chironomidae).
- Preserve a varied landscape with drier and wetter herbaceous meadows and forests, as well as wetlands around the bat habitat.
- Avoid extensive mowing of meadows. Avoid complete mowing at once, keep or install rotational systems which leave part of the grassland or forest borders unmown every year to support overwintering and survival of above-ground immature insect stages of many Lepidoptera and Diptera.
- Avoid simultaneous yearly cutting of forest track margins with tall herb stands.
- Avoid spraying nettles (*Urtica*) with herbicides. This is an important larval host plant for many of the Lepidoptera species that serve as food for *N.leisleri*.
- Preserve trees with woodpecker cavities where *N.leisleri* roosts, as well as standing dead wood.
- Many of the target prey species (Lepidoptera, Ephemeroptera) are attracted by artificial light sources. Use insect friendly street lighting, to reduce the impact on the insects and the bats (Rowse et al. 2015).
- Monitor changes in prey phenology. Climate induced phenological shifts could affect prey availability at the time of highest energy requirement for the bats (birth and lactation).

## Supplementary Material

17B4E480-A37F-5FB7-8FE3-57C3A85957D8 10.3897/BDJ.11.e111146.suppl1 Supplementary material 1 Samples of bat faeces used in this study Data type Sample metadata Brief description Samples of bat faeces collected in this study (Kottenforst, Bonn, Germany, season 2017). When taxonomy analysis for each guano sample retrieved only *N.Leisleri* with 100% BLAST match, the sample was included for further analysis (nine samples marked in bold). File: oo_885015.docx https://binary.pensoft.net/file/885015 Bourlat S.J. et al.

CB08177D-749E-550B-AE8A-6B533300806F 10.3897/BDJ.11.e111146.suppl2 Supplementary material 2 ASV table for the guano samples Data type ASV table Brief description ASV table for the guano samples showing taxonomic assignments for all markers COImldg, COIArt and 16S. All taxonomic assignments represent matches at > 97% identity. File: oo_885016.xlsx https://binary.pensoft.net/file/885016 Bourlat S.J. et al.

E0E75D3A-D525-5CEE-9551-86782C77BE71 10.3897/BDJ.11.e111146.suppl3 Supplementary material 3 Lepidopteran prey species in the guano for all *N.leisleri* samples Data type Relative read abundances Brief description The most frequently found Lepidopteran prey species in the guano for all *N.leisleri* samples, calculated using relative read abundances. All taxonomic assignments represent matches at > 97% identity. File: oo_885018.xlsx https://binary.pensoft.net/file/885018 Bourlat S.J. et al.

F7BEF709-A548-5355-81FE-EC8894A1101F 10.3897/BDJ.11.e111146.suppl4 Supplementary material 4 Arthropod prey species in the guano for all *N.leisleri* samples Data type Relative read abundances Brief description The most frequently found arthropod prey species in the guano for all *N.leisleri* samples, calculated using relative read abundances. All taxonomic assignments represent matches at > 97% identity. File: oo_885019.xlsx https://binary.pensoft.net/file/885019 Bourlat, S.J. et al.

BD5FAB60-C940-5EBC-B6BB-199DB1177DD0 10.3897/BDJ.11.e111146.suppl5 Supplementary material 5 R code for Figures 2-4 Data type R code Brief description R code used to produce Figures 2, 3 and 4. File: oo_885022.txt https://binary.pensoft.net/file/885022 Bourlat S.J. et al.

A42FA9B2-40E7-5A25-831B-BEC14C0A8F40 10.3897/BDJ.11.e111146.suppl6 Supplementary material 6 References used to create Table 3 Data type Text file Brief description References used to create Table 3. File: oo_888222.pdf https://binary.pensoft.net/file/888222 Bourlat S.J. et al.

## Figures and Tables

**Table 1.** Molecular identification of bat (and other mammal) species found in the roosts, detected with metabarcoding of guano pellets. Only samples that had a 100% BLAST match to *N.leisleri* were included in the analysis (in bold). If several species were detected, the samples were excluded from the dietary analyses.

Presumed roost of	sampling date	roost ID	*M.nattereri*	*N.leisleri*	*P.auritus*	*M.bechsteinii*	*M.mystacinus*	*A.flavicollis*
***Nyctalusleisleri***	**26.03.17**	**KF01-01**		**x**				
***Nyctalusleisleri***	**24.05.17**	**KF01-02**		**x**				
***Nyctalusleisleri***	**28.05.17**	**KF01-03**		**x**				
*Nyctalusleisleri*	02.06.17	KF01-04						x
*Nyctalusleisleri*	14.06.17	KF01-05		x		x		
***Nyctalusleisleri***	**23.06.17**	**KF01-06**		**x**				
***Nyctalusleisleri***	**26.06.17**	**KF01-07**		**x**				
***Nyctalusleisleri***	**29.06.17**	**KF01-08**		**x**				
***Nyctalusleisleri***	**08.07.17**	**KF01-09**		**x**				
***Nyctalusleisleri***	**09.08.17**	**KF01-10**		**x**				
***Nyctalusleisleri***	**14.08.17**	**KF01-11**		**x**				
*Nyctalusleisleri*	05.09.17	KF01-12						x
*Nyctalusleisleri*	14.06.17	KF02-01						
*Myotisbechsteinii*	14.06.17	KF02-02						x
*Myotisnattereri*	26.03.17	KF03-01	x	x	x		x	
*Myotisnattereri*	26.06.17	KF03-02				x		
*Myotisbechsteinii*	26.03.17	KF04-01		x				

**Table 2.** Arthropod orders found and number of species in each order in *N.leisleri* guano, all mitochondrial markers combined (COImldg, COIArt and 16S)

**Order/Marker**	**16S**	**mldg**	**COI_Art**	**Total**
**Araneae**	1	0	6	7
**Blattodea**	1	0	0	1
**Coleoptera**	25	6	26	48
**Diptera**	36	7	50	86
**Entomobryomorpha**	0	0	1	1
**Ephemeroptera**	2	5	6	10
**Hemiptera**	20	3	9	28
**Hymenoptera**	0	1	3	4
**Isopoda**	1	1	2	3
**Ixodida**	2	0	0	2
**Lepidoptera**	18	8	106	126
**Lithobiomorpha**	0	0	1	1
**Neuroptera**	7	4	10	15
**Opiliones**	2	0	1	3
**Psocoptera**	1	0	6	6
**Siphonaptera**	1	0	0	1
**Trichoptera**	2	1	14	16
**Total**	119	36	241	358

**Table 3.** Ecological characteristics of the most abundant Lepidopteran species found in the bat guano, based on RRA. References to create the table can be found in Suppl. material 6.

Order	Family	Genus, species	Wingspan (mm)	Larval food	Flying time	Number of generations each year	Habitat
Lepidoptera	Noctuidae	*Apameaunanimis*	29-38	Poaceae, mainly *Phalarisarundinacea* and *Phragmitesaustralis*	May-July	1	Moist areas, including wetlands, riparian forests, wet meadows and stream or ditch margins
Lepidoptera	Tortricidae	*Cydiafagiglandana*	12-16	*Fagus*, *Quercus*, *Castaneasativa*, in the seeds	April-September	1	Forests, woodlands, parks, hedgerow trees, isolated trees
Lepidoptera	Geometridae	*Xanthorhoeferrugata*	18-22	*Galium*, *Stellaria*, *Campanula*, *Cirsium*	April-September	2	Shrublands, fringes, forest edges, forest roads, and other mostly woody habitats
Lepidoptera	Tortricidae	*Cnephasiaasseclana*	15-18	A wide range of herbaceous plants	June-August	1	Open woodlands, scrub, hedgerows, grasslands, gardens
Lepidoptera	Erebidae	*Hypenaproboscidalis*	25-38	Largely *Urticadioica*, but also *Humulus*, *Stachys*, *Aegopodium*	May-September	2	Areas with nettles in deciduous, non-deciduous, mixed forests and gardens
Lepidoptera	Noctuidae	*Axyliaputris*	30-36	Many including *Urtica*, *Trifolium*, *Triticum*, *Polygonum*, *Rumex*, *Medicago*	April-September	2	Herbaceous meadows, hedges and bushes, stream banks and ditches, fens, deciduous and mixed forests, orchard meadows, gardens, parks
Lepidoptera	Pyralidae	*Dioryctriaabietella*	27-33	*Abies*, *Picea*, *Larix*, *Pinus*, shoots and cones	May-October	2	Coniferous and mixed forests, parks
Lepidoptera	Noctuidae	*Oligiaversicolor*	24-28	*Carex*, *Poa*, *Luzula*, *Bracylipodium*	June-August	1	Wet meadows, bogs, wet heaths, forest marshes
Lepidoptera	Noctuidae	*Eupsiliatransversa*	40-48	*Populus*, *Salix*, *Corylus*, *Fagus*, *Quercus*, *Ulmus*, *Malus*, *Crataegus*, *Rubus*, *Prunus* and others	August-November, February-April	1	Dry to moist deciduous and mixed forest, hedges, bushland, orchard meadows, gardens, parks
Trichoptera	Lepidostomatidae	*Lepidostomahirtum*	14-20	Scraper and shredder of algae and vegetation	June-September	1	Larvae in running water and littoral zones of standing waters
Ephemeroptera	Polymitarcyidae	*Ephoronvirgo*	20-32	Filter feeders in sediment	July-September	1	Nymphs in the lower stretches of midsized and larger rivers
Ephemeroptera	Ephemeridae	*Ephemeradanica*	35-45	Filter feeders in gravel	April-September(main season in May-June)	1	Nymphs in clear water rivers and lakes
Ephemeroptera	Caenidae	*Caenishoraria*	8-12	Filter feeders in mud and silt	May-September	1	Nymphs in pools and margins of rivers, canals and streams or in lakes and ponds
Diptera	Fanniidae	*Fannialeucosticta*	5-7	Rotting plant material, compost, carrion, dung	June-September	?	Larvae in rotting plant material, compost, garbage, bat roosts, bird nests
Diptera	Tipulidae	*Tipulalunata*	~ 40	Plant roots	April-July	1	Larvae mainly in the soil and in the litter layer of forests and shrubs or under moss cushions
Diptera	Limoniidae	*Cheilotrichiacinerascens*	12-16	Dead *Fagus* leaves	May-October	1	Larvae in the leaf litter in wetter beech forests, in swamps and marshes
