# Supplementary material for: Metabarcoding dietary analysis in the insectivorous bat Nyctalusleisleri and implications for conservation
Source: Biodivers Data J. 2023 Nov 14;11:e111146. doi: 10.3897/BDJ.11.e111146 (PMC10838074; doi:10.3897/BDJ.11.e111146)
Supplement: Supplementary material 1 — Samples of bat faeces used in this study [file bdj-11-e111146-s001.docx]

**Supplementary table 1:** Samples of bat feces collected in this study (Kottenforst, Bonn, Germany, season 2017). When taxonomy analysis for each guano sample retrieved only *N. Leisleri* with 100% BLAST match, the sample was included for further analysis (9 samples marked in bold).

| **Presumed roost of** | **Sample ID** | **date** | **No of pellets** | **Isopropanol** | **Silica** |
| --- | --- | --- | --- | --- | --- |
| ***Nyctalus leisleri*** | **KF01-01** | **26.03.2017** | **20** | **x** | **x** |
| ***Nyctalus leisleri*** | **KF01-02** | **24.05.2017** | **30** | **x** | **x** |
| ***Nyctalus leisleri*** | **KF01-03** | **28.05.2017** | **3** | **x** | **x** |
| *Nyctalus leisleri* | KF01-04 | 02.06.2017 | 25 | x | x |
| *Nyctalus leisleri* | KF01-05 | 14.06.2017 | 50 | x | x |
| ***Nyctalus leisleri*** | **KF01-06** | **23.06.2017** | **28** | **x** | **x** |
| ***Nyctalus leisleri*** | **KF01-07** | **26.06.2017** | **25** | **x** | **x** |
| ***Nyctalus leisleri*** | **KF01-08** | **29.06.2017** | **30** | **x** | **x** |
| ***Nyctalus leisleri*** | **KF01-09** | **08.07.2017** | **50** | **x** | **x** |
| ***Nyctalus leisleri*** | **KF01-10** | **09.08.2017** | **>100** |  | **x** |
| ***Nyctalus leisleri*** | **KF01-11** | **14.08.2017** | **30** |  | **x** |
| *Nyctalus leisleri* | KF01-12 | 05.09.2017 | >200 |  | x |
