# Supplementary material for: Metabarcoding dietary analysis in the insectivorous bat Nyctalusleisleri and implications for conservation
Source: Biodivers Data J. 2023 Nov 14;11:e111146. doi: 10.3897/BDJ.11.e111146 (PMC10838074; doi:10.3897/BDJ.11.e111146)
Supplement: Supplementary material 6 — References used to create Table 3 [file bdj-11-e111146-s006.pdf]

References for table 3:

Bauernfeind E, Soldán T. 2012. The Mayflies of Europe, Apollo Books, Stenstrup. 781pp. Ebert G. (Ed.) 1997. Die Schmetterlinge Baden-Württembergs, Vol. 5. Nachtfalter III. Ulmer, Stuttgart (Hohenheim) 1997. Brown JW, Robinson G, Powell JA. 2008. Food plant database of the leafrollers of the world (Lepidoptera: Tortricidae) (Version 1.0). http://www.tortricid.net/foodplants.asp. Ebert G. (Ed.) 1997. Die Schmetterlinge Baden-Württembergs, Vol. 6. Nachtfalter IV. Noctuidae 2. Teil. Ulmer, Stuttgart (Hohenheim). Heiß R, Flügel H-J. 2015. Die Schnaken auf dem Gelände des Lebendigen Bienenmuseums (Diptera: Tipulidae). Philippia 16, 267-281. Kureck A, Fontes RJ 1996. The life cycle and emergence of Ephoron virgo, a large potamal mayfly that has returned to the River Rhine. Arch. Hydrobiol. Suppl. (Large Rivers 10), 113, 319-323. Malicky H. 2004. Atlas of European Trichoptera. Second Edition. Springer, Dordrecht. Rozkošný R, František G, Pont AC. 1997. The European Fanniidae (Diptera). Acta Scientarium Naturalium Academiae Scientiarum Bohemicae Brno, 31, 1-80. Steiner A, Ebert G. 1998. Die Schmetterlinge Baden-Württembergs, Vol. 7. Nachtfalter V. Noctuidae 3. Teil. Ulmer, Stuttgart (Hohenheim). Steiner A, Ratzel U, Top-Jensen M, Fibiger M. 2014. Die Nachtfalter Deutschlands. BugBook Publishing, Østermarie, 878pp. Stirling P, Parsons M. 2012. Field Guide to the Micromoths of Great Britain and Ireland. British Wildlife Publishing, Gillingham, 416pp. Zilli A, Ronkay L, Fibiger M 2005. Apameini. In: W. G. Tremewan (eds): Noctuidae Europapaeae, Vol. 8. Entomological Press, Sorø, 127pp
